# *Bartonella* in bat flies from the Egyptian fruit bat in the Middle East

**DOI:** 10.1007/s00436-024-08165-6

**Published:** 2024-02-27

**Authors:** Eva Špitalská, Martin Ševčík, Yevheniy-Yuliy Peresh, Petr Benda

**Affiliations:** 1 Institute of Virology, Biomedical Research Center, Slovak Academy of Sciences, Dúbravská Cesta 9, 845 05 Bratislava, Slovakia; 2 Department of Zoology, Faculty of Science, Charles University, Viničná 7, 128 43 Praha 2, Czech Republic; 3 Department of Zoology, National Museum (Natural History), Václavské nám. 68, 115 79 Praha 1, Czech Republic

**Keywords:** *Bartonella*, *Eucampsipoda aegyptia*, *Rousettus aegyptiacus*, Palaearctic, Middle East

## Abstract

In the family of fruit bats, Pteropodidae Gray, 1821, as in the third most diverse group of bats (Chiroptera), the bacterium of the genus *Bartonella* was detected in several species as well as in a few species of their insect ectoparasites in some tropical and sub-tropical regions of the Old World. The Egyptian fruit bat, *Rousettus aegyptiacus* (Geoffroy, 1810), is one of the most widespread fruit bats, occurring between South Africa, Senegal, and Pakistan. In this bat species, *Candidatus* Bartonella rousetti has been detected in three African populations in Nigeria, Kenya, and Zambia. This fruit bat, however, also occurs in the Palaearctic, an area isolating the species geographically and phylogenetically from the Afrotropical part of its distribution range. We screened the blood-sucking bat flies (family Nycteribiidae) from *R. aegyptiacus* for the presence of the *Bartonella* bacteria. A rich material of bat fly *Eucampsipoda aegyptia* (Macquart, 1850), a monoxenous ectoparasite of the Egyptian fruit bats, was collected at 26 localities in seven countries (Egypt, Iran, Jordan, Lebanon, Oman, United Arab Emirates, and Yemen) of the Middle East in 2007–2013. The DNA isolates from the bat flies were subjected to a three-marker (*gltA*, *ssrA*, and intergenic spacer region, ITS) multilocus sequence analysis. Based on the amplification of the fragment of *ssrA* gene by a real-time PCR, 65 *E*. *aegyptia* samples from 19 localities in all seven countries were positive for the bacteria. One to five *Bartonella*-positive individuals of *E*. *aegyptia* were collected per one individual of *R. aegyptiacus*. An analysis of the ITS and *gltA* genes indicated the presence of an uncultured *Bartonella* sp., belonging to the *Cand.* B. rousetti genogroup, identified from populations of the Egyptian fruit bat in Africa. These results support the hypothesis that *Bartonella*’s diversity corresponds to its host’s diversity (and phylogenetic structure). Specific lineages of pathogens are present in specific phylogenetic groups of bats.

**Supplementary Information:**

The online version contains supplementary material available at 10.1007/s00436-024-08165-6.

## Introduction

The genus *Bartonella* Strong, Tyzzer, Brues et Sellards, 1915 (Hyphomicrobiales: Bartonellaceae) comprises phylogenetically diversified facultative intracellular Gram-negative α-proteobacteria that infect mainly the erythrocytes and endothelial cells of mammals (Eicher and Dehio 2012). These bacteria are distributed worldwide and transmitted predominantly by blood-feeding arthropods (Chomel et al. 2009).

McKee et al. (2021) suggested bats (Chiroptera) are a group of mammals that have a crucial role in the origin and spread of the *Bartonella* bacteria among geographical regions and other mammal groups. In bats, several taxa of blood-feeding arthropods can be found, which can help in the dispersal of this bacterium (Marshall 1982; Szubert-Kruszyńska and Postawa 2008). The available results demonstrated that the genus *Bartonella* used to be found most frequently in one group of these arthropods, in the family of bat flies, Nycteribiidae (Szentiványi et al. 2019). Identical genotypes of *Bartonella* are often reported from bats and their bat flies, which suggest that bat flies act as vectors for the spreading of this bacterium among bats and perhaps also other mammals (cf. Kamani et al. 2014; Brook et al. 2015; Moskaluk et al. 2018; Judson et al. 2015; Dietrich et al. 2016; Qiu et al. 2020).

In the last decade, several new strains/genotypes of *Bartonella* were detected in bats and their flies throughout the world (see Han et al. 2022: 2, Fig. 1). However, the taxonomic diversity of *Bartonella* is only poorly manifested among bat taxa and/or populations. Due to this, our understanding of whether and how particular species of *Bartonella* are shared among related bat species is somewhat limited.

**Fig. 1:**
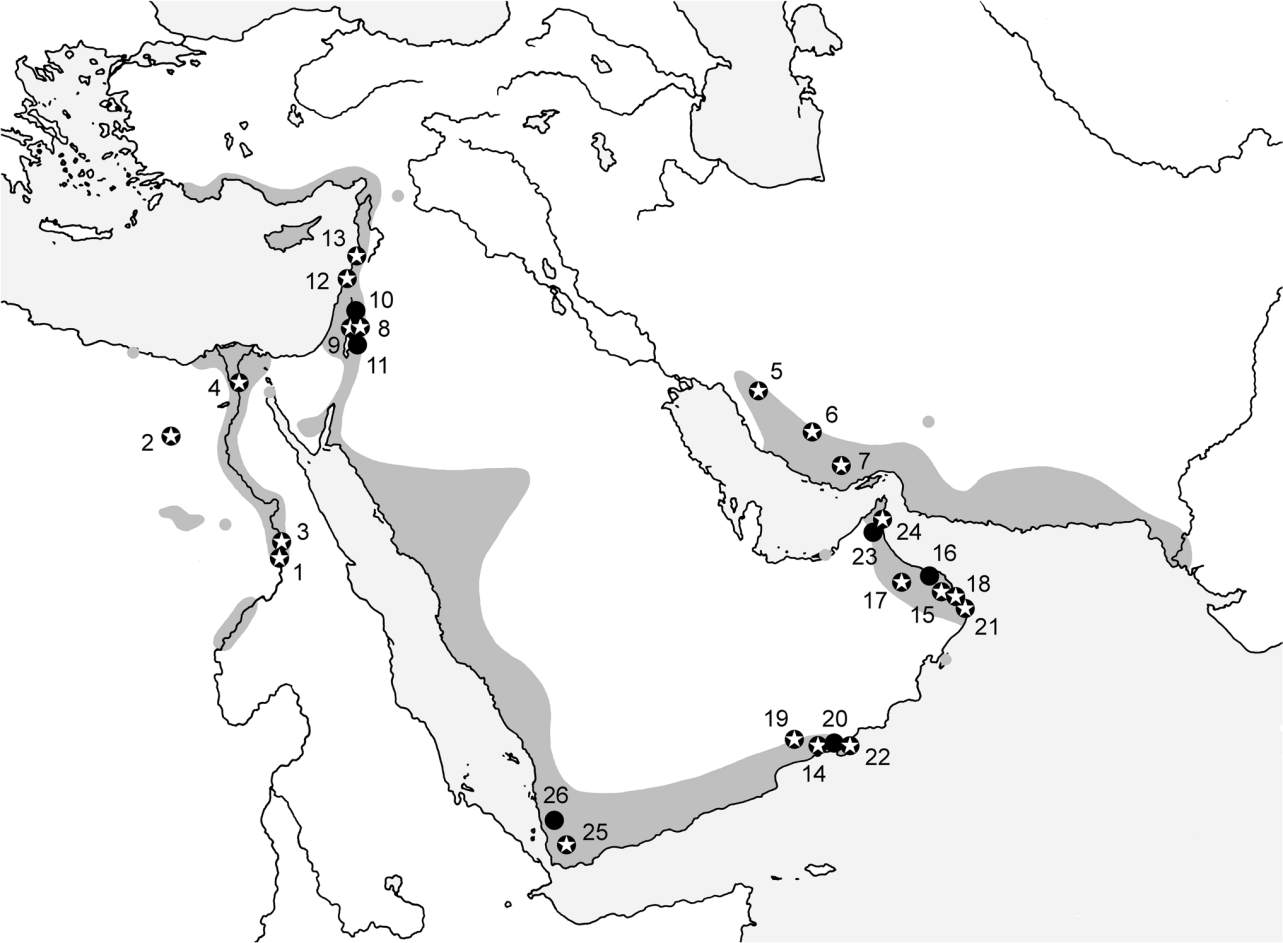
A map of the bat fly *Eucampsipoda aegyptia* collection sites used in this study (circles); a circle with an asterisk marks the pathogen presence, and the numbers correspond with locality numbers in Material and methods. The dark grey area shows the distribution range of *Rousettus aegyptiacus* in the Middle East reconstructed after Benda et al. (2011, 2023)

The family of fruit bats, Pteropodidae Gray, 1821, is one of the richest bat groups concerning species diversity, broadly distributed in the tropics and subtropics of the Old World. Despite this, reports on the prevalence of *Bartonella* in these bats and their bat flies are available only from a few localities (Bai et al. 2018; Kamani et al. 2014; Dietrich et al. 2016; Brook et al. 2015; Wilkinson et al. 2016; Qiu et al. 2020; Fagre et al. 2023). The Egyptian fruit bat, *Rousettus aegyptiacus* (Geoffroy, 1810), ranks among fruit bats with extensive geographical distribution—it is the only fruit bat species occurring in two continents; it lives in most of Africa and southwestern Asia. It occupies areas around the Gulf of Guinea from Senegal to Angola, in southern and eastern Africa from the Cape to Eritrea, and the southwest part of the Palaearctic, from Egypt, Yemen, and Turkey to Pakistan (Kwiecinski and Griffiths 1999; Benda et al. 2011). The distribution range in the Middle East (comprising wholes or parts of Egypt, Sudan, Turkey, Cyprus, Levant, Arabia, Iran, and Pakistan, see Benda et al. 2011 for a review, with additions by Benda et al. 2012, 2023, Judas et al. 2018, Benda and Ševčík 2020) is an occurrence spot isolated both geographically and phylogenetically from the Afro-tropical populations (Střibná et al. 2019).

The search for *Bartonella* in the populations of *Rousettus aegyptiacus* brought detection of the genotype *Candidatus* Bartonella rousetti from the bat fly *Eucampsipoda africana* Theodor, 1955, a frequent ectoparasite of this fruit bat. This strain was found in this bat fly collected in Nigeria (Bai et al. 2018) and Zambia (Szentiványi et al. 2022) and was also confirmed in the blood of *R*. *aegyptiacus* from Kenya (Kosoy et al. 2010). The knowledge of *Bartonella* in the Egyptian fruit bat is thus limited to a few sites in tropical Africa. At the same time, this pathogen has not been screened in the Palaearctic populations.

The absence of any evidence of *Bartonella* in the Palaearctic populations of the Egyptian fruit bat and its monoxenous arthropod parasite was an impulse for a more detailed study of the bat fly *Eucampsipoda aegyptia* (Macquart, 1850), an obligatory ectoparasite of *Rousettus aegyptiacus*. The screening for the presence of *Bartonella* was done in almost the entire known distribution range of this bat fly in the Middle East, from Egypt to Iran. Our study finally brought new evidence of the *Bartonella* presence from a large part of the Palaerctic range of the Egyptian fruit bat and, thus, of its wide distribution and relatively common occurrence.

## Material and methods

### The material examined

Our study comprises the populations of the Egyptian fruit bat, *Rousettus aegyptiacus*, from the Middle East, including its NE African part. The bat flies *Eucampsipoda aegyptia* were collected from the fruit bats at the following 26 sites situated in seven countries (Fig. 1): Egypt: (1) Aswan, 24°07′N, 32°54′E, 92 m a. s. l. (24 January 2010, 10 January 2011); (2) Bahariya Oasis, Bawiti, 28°21′N, 28°52′E, 98 m a. s. l. (18 January 2010, 30 December 2010, 2 January 2011); (3) El A’aqab, 24°16′N, 32°54′E, 96 m a.s.l. (25 January 2010); (4) El Qahirah, Gezira Island, 30°03′N, 31°13′E, 20 m a. s. l. (29 January 2010); Iran: (5) Bishapur, Sasan Cave, 29°47′N, 51°35′E, 860 m a. s. l. (6 October 2011); (6) Jahrom, Sang Eshkan, 28°29′N, 53°35′E, 1102 m a. s. l. (8 October 2011); (7) Zangard, 27°13′N, 54°38′E, 493 m a. s. l. (9 October 2011); Jordan: (8) Iraq Al Amir, Wadi As Sir, 31°55′N, 35°45′E, 515 m a. s. l. (2 July 2010); (9) Jufat Al Qafrayn, 31°53′N, 35°37′E,  235 m a. s. l. (15 July 2010); (10) Nahla, 32°17′N, 35°51′E, 728 m a. s. l. (13 July 2010); (11) An Nuzha, Wadi Al Wala, 31°33′N, 35°44′E, 335 m a. s. l. (11 July 2010); Lebanon: (12) Dahr El Mghara, Aaonamie Cave, 33°40′N, 35°27′E, 255 m a. s. l. (19 January 2008); (13) Trablous, Matal El Azraq Cave, 34°25′N, 35°50′E, 15 m a. s. l. (21 January 2007); Oman: (14) Ain Sahalnawt, 17°09′N, 54°11′E, 123 m a. s. l. (27 March 2012); (15) Al War, Wadi Khabbah, 22°56′N, 58°51′E, 406 m a. s. l. (5 April 2011); (16) Bidbid, Wadi Dabaum, 23°25′N, 58°08′E, 205 m a. s. l. (26 March 2011); (17) Misfah, 23°14′N, 57°08′E, 1196 m a. s. l. (9 April 2011); (18) Mithqub, Wadi Harabein, 23°04′N, 58°59′E, 52 m a. s. l. (2 November 2009); (19) Mudhai, 17°29′N, 53°21′E, 542 m a. s. l. (25 March 2012); (20) Shihayt, Wadi Darbat, 17°09′N, 54°28′E, 326 m a. s. l. (28 March 2012); (21) Tayma, 22°31′N, 59°20′E, 196 m a. s. l. (3 April 2011); (22) Wadi Hannah, 17°03′N, 54°37′E, 310 m a. s. l. (30 March 2012); United Arab Emirates: (23) Al Khari, Shawka Dam, 25°06′N, 56°03′E, 295 m a. s. l. (29 October 2013); (24) Shis, 25°17′N, 56°15′E, 363 m a. s. l. (30 October 2013); Yemen: (25) Mashgab, Ash Shamshara, 13°21′N, 43°57′E, 1170 m a. s. l. (26 October 2007); (26) Wadi Zabid, Al Mawkir, 14°10′N, 43°30′E, 270 m a. s. l. (30 October 2007).

The Egyptian fruit bats were caught using standard methods like mist or hand nets. The bats’ whole bodies were checked for the presence of ectoparasites. The bat flies’ maximum individuals were removed, collected using tweezers, and preserved in 96% ethanol.

The fixed bat fly specimens were studied with a microscope without additional interventions. The species and sex determinations of the *Eucampsipoda aegyptia* specimens were carried out using the key by Theodor (1967: 413–416). The taxonomy and nomenclature follow Maa (1965).

Only a part (176/196) of the collected bat fly material was used to detect pathogens. The examined samples of extracted DNA are stored at the Institute of Virology, Biomedical Research Center, Slovakian Academy of Sciences, Bratislava, Slovakia. The other bat fly *E. aegyptia* material not screened for a pathogen presence remains in a private collection of Martin Ševčík, Nitra, Slovakia.

### DNA extraction, molecular genetic, and statistical analyses

The ethanol-fixed bat fly specimens were washed with sterile water, dried, and crushed with a sterile scalpel. Following the manufacturer’s protocol, their DNA was extracted using the QIAamp DNA Mini kit (Qiagen). Thirty additional samples from Egypt were stored as dry specimens, and their DNA was extracted by chelex (Walsh et al. 1991). The quantity and quality of the DNA were assessed by Nano Photometer Pearl (Implen, Germany), and the extracted DNA was used as a template for the PCR amplification to determine the presence of *Bartonella*, with the following species identification (Table 1). The *Bartonella* positive amplicons were purified and then analyzed by sequencing in both directions with the same primers as for the PCR amplification by Macrogen Inc. (Amsterdam, The Netherlands). The obtained partial sequences of ITS and *gltA* genes were compared with those available in the GenBank using the Basic Local Alignment Search Tool (BLAST; http://blast.ncbi.nlm.nih.gov).

**Table 1:** Primers used for the detection of the *Bartonella* presence in the examined bat flies collected from the Egyptian fruit bats

Primer name	Primer sequence (5’–3’)	Target gene	A. g. (bp)	A. t. (°C)	Reference
ssrA-R1	AAG GCT TCT GTT GCC AGG YG	*ssrA*	124	56.6	Mardosaitė-Busaitiene et al. (2019)
ssrA-F1	AGT TGC AAA TGA CAA CTA TGC GG				
ssrA-P1	FAM-ACC CCG CTT AAA CCT GCG ACG GTT				
BA325s	CTT CAG ATG ATG ATC CCA AGC CTT CTG GCG	16S–23S rRNA gene ITS region	420–780	66	Maggi et al. (2009)
BA1100as	GAA CCG ACG ACC CCC TGC TTG CAA AGC A				
BhCS 781p	GGG GAC CAG CTC ATG GTG G	*gltA*	357	43	Norman et al. (1995)
BhCS 1137n	AAT GCA AAA AGA ACA GTA AACA				

*A. g. (bp)* Amplicon gene; *A. t. (°C)* annealing temperature

Phylogenetic analyses were conducted in MEGA11 (Tamura et al. 2021) and the phylogenetic tree based on ITS region was constructed using the neighbor-joining method (Saitou and Nei 1987) with the Kimura 2-parameter method (Kimura 1980). Partial *gltA* genes and ITS region sequences for representative samples were submitted to the GenBank under the accession numbers OR553951–OR553952 for the *gltA* gene, and OQ058984–OQ058989 and OR523867–OR523871 for the ITS region, respectively.

Statistical analyses testing the geographical and sexual differences in the presence of *Bartonella* species in *Eucampsipoda aegyptia* specimens have been done using Fisher’s exact test with an online calculator (http://www.socscistatistics.com). The *p* value < 0.05 was considered as proof of significant difference, and 95% confidence intervals (CI) were calculated using an online calculator (http://epitools.ausvet.com.au).

## Results

### Bat flies, localities, and *Bartonella* presence

Altogether, 176 individual bat flies *Eucampsipoda aegyptia* were analyzed for the presence of *Bartonella*; these flies were collected from 68 individuals of *Rousettus aegyptiacus* (Egypt: 4 sites, 44 flies; Iran: 3 sites, 37 flies; Jordan: 4 localities, 13 flies; Lebanon: 2 sites, 5 flies; Oman: 9 sites, 68 flies; UAE: 2 sites, 5 flies; Yemen: 2 sites, 4 flies). Based on the real-time PCR analysis, a total of 65 of the bat fly DNA samples (36.9% of the 176 samples analyzed; 95% confidence interval (next CI) 29.80–44.06) were found positive for *Bartonella* (Table 2), and further characterized. The *Bartonella* DNA was found both in females (40.91%; 95% CI 29.05–52.77) and males (34.55%; 95% CI 25.66–43.43). The difference between the presence depending on sex was not significant (*p* = 0.423). The *Bartonella-*positive bat flies were collected from 37 individuals of *R. aegyptiacus*, with a frequency between one and five positive bat flies per fruit bat individual. The DNA of *Bartonella* spp. was detected in the samples in all countries from where the samples were examined (Table 2).

**Table 2:** Total numbers of the analyzed *Eucampsipoda aegyptia* and the *Bartonella* positive samples ( +)

Country	♀♀	♂♂	Total	♀♀ +	♂♂ +	Total +	% positive
Egypt	5	9	14	2	6	8	57.1
Egypt (dry)	12	18	30	3	7	10	33.3
Jordan	7	6	13	1	3	4	30.8
Lebanon	4	1	5	4	0	4	80.0
UAE	3	2	5	0	1	1	20.0
Yemen	1	3	4	1	2	3	75.0
Oman	19	49	68	9	12	21	30.9
Iran	15	22	37	7	7	14	37.8
total	66	110	176	27	38	65	36.9

### Genetic diversity

**Fig. 2:**
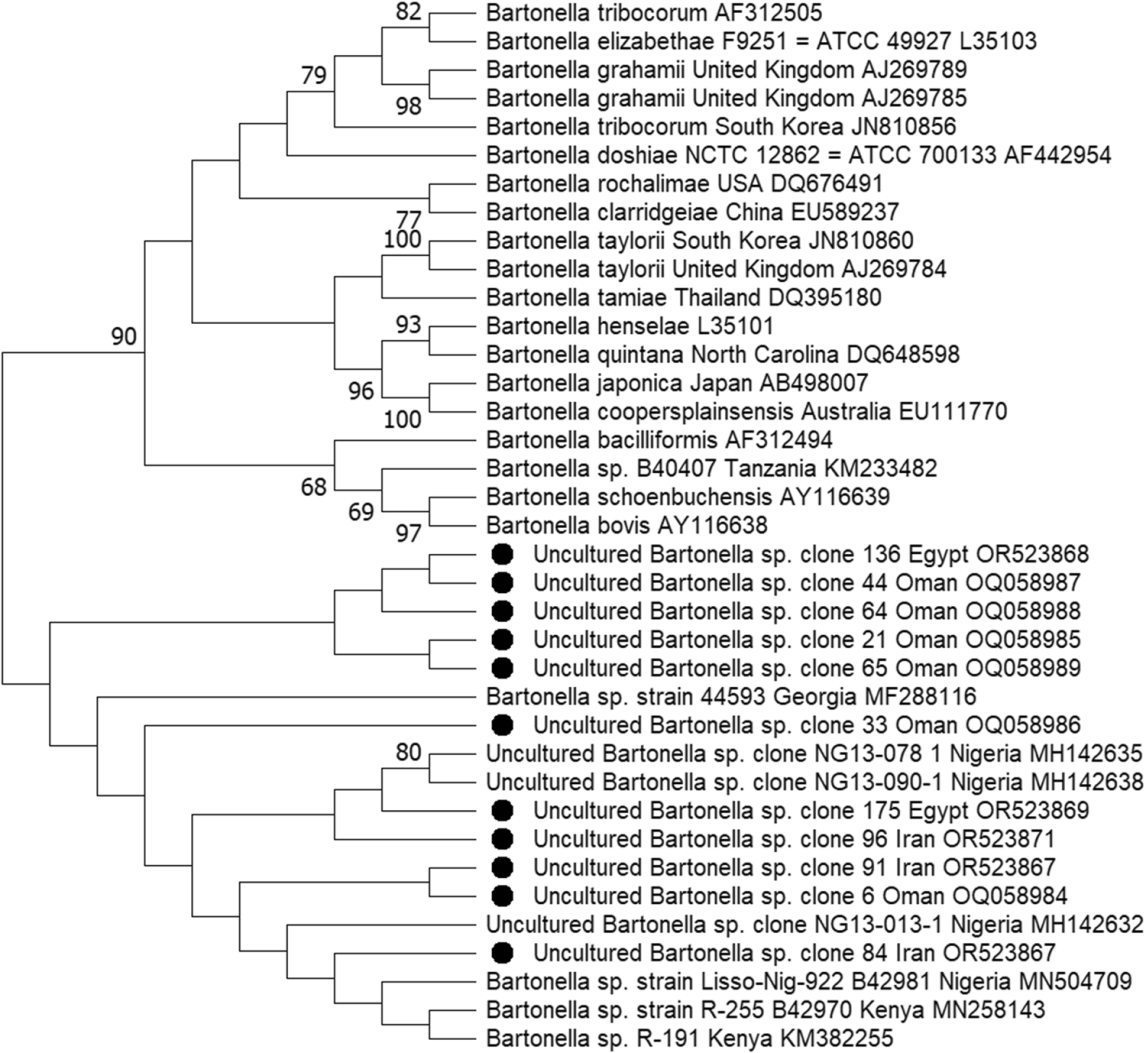
Phylogenetic relationship of *Bartonella* strains based on the internal transcribed spacer sequences (ITS). The neighbour-joining method by the Kimura 2-parameter distance and bootstrap calculation was conducted with 500 replicates for phylogenetic analysis. GenBank Accession Numbers are provided for all sequences

Fifty-four good-quality sequences of fragments of the ITS region (*n* = 39) and *gltA* (*n* = 15) gene were obtained and analyzed. The sequence analysis of the partial ITS region of all 39 samples revealed the presence of *Bartonella* strains of the *Ca.* B. rousetti genogroup (Fig. 2). Twenty samples (4 females from 4 bats from Lebanon; 1 female and 2 males from 1 bat from Yemen; 2 males from 2 bats from Jordan; 3 males from 3 bats from Egypt; 4 females and 4 males from 5 bats from Iran) were identical with the uncultured *Bartonella* clone 84 deposited in the GenBank (OR523867)— > 99% sequence identity to *Bartonella* sp. R-191 (KM382255) was detected in the blood of *Rousettus aegyptiacus* from Kenya (Kosoy et al. 2010). *Bartonella rousetii* is a name for *Bartonella* sp. R-191 proposed by Bai et al. (2018). Another four male samples of bat flies collected from *R. aegyptiacus* in Jordan, UAE, Egypt, and Iran, were identical with the uncultured *Bartonella* clone 136 deposited in the GenBank (OR523868)— > 96% sequence identity to *Ca.* B. rousetti (KM382255). Other five samples of *Eucampsipoda aegyptia*, 1 male and 2 females from 1 bat from Egypt, and 1 male and 1 female from 1 bat from Iran were identical to the uncultured *Bartonella* clone 175 deposited in the GenBank (OR523869)— > 97% sequence identity to *Ca.* B. rousetti (KM382255). Sequences from 1 male and 1 female from 2 bats from Iran were identical to the uncultured *Bartonella* clone 91 deposited in the GenBank (OR523870)— > 98% sequence identity to *Ca.* B. rousetti (KM382255). The *Bartonella* ITS region sequences derived from 7 individuals of *E. aegyptia*, 3 males (OQ058984–OQ058986) and 3 females (OQ058987–OQ058989) from Jordan, and 1 female from Iran (OR523871) shared > 94% similarity with *Ca.* B. rousetti (KM382255).

In total, 15 bat flies positive for the *gltA* gene were further analyzed, and the analysis revealed two types of the *gltA* sequences; sequence type 1 (OR553951) from a male of *Eucampsipoda aegyptia* collected from the fruit bat from Egypt showed 99% identity to two clones, the uncultured *Bartonella* clone YNBS/BF03 (OP433671) and clone YNBS/BF06 (OP433673) identified previously from *E. africana* from China (Kuang et al. 2022), and 85% identity to *Ca.* B. rousetti (HM363764). The maximum likelihood tree of the genus *Bartonella* based on the *gltA*, *ftsZ*, and *rpoB* genes using the MLSA approach showed *Bartonella* strain R-191 closely clustered with the fly-associated strain YNBS/BF03 (Kuang et al. 2022).

In total, 14 DNA samples of *Bartonella* of the gltA genotype 1 were identical, and their ITS regions represented three sequences (OQ058984, OR523870, OR523871). They originated from 3 females of *E. aegyptia* collected from 2 fruit bats from Oman, four samples of *E. aegyptia* from Iran, three from Lebanon, and one from Yemen. The nucleotide sequences of the *gltA* genotype 2 originated from a female of *E. aegyptia* collected from a fruit bat from Jordan (OR553952) showed 96% identity to *Bartonella* clone Batfly-3 (LC461051) previously found in *E. africana* from Zambia (Qiu et al. 2020). The sequence of the ITS region from this bat fly was unusable. For the comparison of *Bartonella* sequences amplified from *E. aegyptia* with selected sequences obtained from the GenBank via the BLAST query, see Table S1.

## Discussion

Our study presents screening results for *Bartonella* presence in one of the obligatory parasitic species of Egyptian fruit bat colonies in the Middle East. We detected the presence of *Bartonella* sp. in the bat fly *Eucampsipoda aegyptia* collected from *Rousettus aegyptiacus* in this region for the first time. Moreover, we found this bacterium to be distributed over almost the whole Palaearctic range of the Egyptian fruit bat, except for the northernmost countries of the range, like Turkey, Cyprus, and Syria.

The analysis of the ITS region revealed the presence of eleven strains of *Bartonela* belonging to the genogroup *Ca.* B. rousetti. Twenty positive samples coming from a large area comprising Egypt, Lebanon, Jordan, Yemen, and Iran showed almost hundred percent identity to *Bartonella* R-191 that was identified in the Egyptian fruit bat population in Africa, i.e., in the blood samples of *R. aegyptiacus* from Kenya and the bat fly *Eucampsipoda africana* collected from the same species in Nigeria (Kosoy et al. 2010; Bai et al. 2018). The remaining detected sequences of the ITS region were also similar to this region of *Ca.* B. rousetti, and our strains of uncultured *Bartonella* were similar to the uncultured *Bartonella* clones also identified in the African populations of the Egyptian fruit bat, from the samples of *Eucampsipoda africana* collected from these bats in Nigeria (Bai et al. 2018). On the other hand, the analysis of the partial *gltA* gene sequences of uncultured *Bartonella* sp. from our study showed that they are identical to the uncultured *Bartonella* clones identified in *Eucampsipoda africana* from Yunnan, China (Kuang et al. 2022).

These results correspond with the results of previous studies, which demonstrated that monoxenous parasites, like *Eucampsipoda aegyptia*, express a lower diversity of infection by *Bartonella* over polyxenous species but a higher prevalence of these bacteria (Sándor et al. 2018). Simultaneously, they support the hypothesis that several very similar lineages of *Bartonella* occur in different geographical regions. Thus, the range size of the host distribution cannot be the main drive of *Bartonella* diversification. Hence, *Bartonella*’s diversity corresponds to its host’s diversity (and phylogenetic structure). Specific lineages of pathogens are present in specific phylogenetic groups of bats (species groups, families, etc.; cf. McKee et al. 2016). Besides this, the increasing phylogenetic distances among hosts decrease the probability of the pathogen transfers between them (McKee et al. 2016). This suggests that the determinant of the *Bartonella* distribution is its hosts’ diversity rather than its geographical distribution. On the other hand, evidence of *Bartonella* transfer between phylogenetically distant species increases (including that between wild and domestic animals; Frank et al. 2018).

The presence of *Bartonella* was surveyed in the bat flies *Eucampsipoda aegyptia*, whose distribution range corresponds closely with that of its primary host, *Rousettus aegyptiacus*. This bat fly is a monoxenous parasite (see above) whose life cycle occurs on the host’s body or in its proximity. Therefore, the parasitation of any other host species is exceptional or rather excluded (see Kock and Nader 1979). This close relation explains the presence of *Bartonella* in the colonies of *R. aegyptiacus*, but the role of the insect parasite in the spread of *Bartonella* remains to be elucidated. Numerous authors (Morse et al. 2012a, b; Dick and Dittmar 2014; Olival et al. 2015; Wilkinson et al. 2016; Han et al. 2017) suggested that bat flies of the family Nycteribiidae act as vectors of the *Bartonella* bacteria. Some of them (Olival et al. 2015; Wilkinson et al. 2016) suggested that bat flies act as reservoirs of these bacteria. McKee et al. (2021) hypothesized that *Bartonella* species evolved from symbionts found in blood-feeding ectoparasites because these arthropods depended on symbionts for additional nutrients (Husnik 2018). Bat flies are obligate ectoparasites of bats and contain endosymbiotic prokaryotes whose role is poorly understood. However, they are assumed to establish a symbiotic relationship with mutualistic bacteria (Morse et al. 2013). The blood-sucking dipterans of the superfamily Hippoboscoidea require milk secretion for larval development, and certain bacteria, such as *Bartonella* and *Wolbachia*, can be vertically transmitted during this process. These bacteria can also be transmitted horizontally through parasitoids or contact with contaminated saliva (de Bruin et al. 2015; Heath et al. 1999). However, horizontal transmission has not been recorded in the nycteribiid bat flies or other hippoboscoids.

Our study unveils Bartonella’s geographical distribution and genetic diversity within the Palaearctic population of *Rousettus aegyptiacus* in its almost complete range. However, the calculation of prevalency that could indicate some aspects of *Bartonella* biology remains omitted. The reason is that it requires a different way of data and material collection than was used in our study, i.e., to be taken from several colonies during and throughout the year. Although the biology of *Eucampsipoda aegyptia* is partly known from Egypt (Hafez et al. 1978), these data are insufficient regarding the range size and habitat diversity of the Middle East. Besides the detailed description of the *Eucampsipoda* life cycle and biology in various segments of its distribution range, for the complex picture of *Bartonella* biology, it is also necessary to monitor the bat host, consider the pathogen outbreak and other details like the changes in colony behavior or roost switches. So, extensive additional research is still necessary to describe the complete role of bat flies in the *Bartonella* transfer and its biology in bats.

In a recent study, antibodies against bat-associated *Ca.* B. rousetti were detected in humans (Bai et al. 2018). It indicates that bat-associated bacteria can potentially infect humans. However, antibodies against *Bartonella* tend to be highly cross-reactive within the genus and with other non-*Bartonella* agents. The DNA of *Bartonella* sp. was detected in the bat saliva, urine, and guano (Dietrich et al. 2017; Veikkolainen et al. 2014). Thus, the possibility of transmission of *Bartonella* to humans does not represent a direct and natural way.

On the other hand, even an accidental visit to a fruit bat roost could be potentially dangerous. Since the Egyptian fruit bat frequently uses anthropogenous roosts, it thus remains in close contact with humans. Eventually, humans could represent one of the connecting links of the bacterium transmission and spread.

### Supplementary Information

Below is the link to the electronic supplementary material.

Supplementary file1 (DOCX 21 KB)

## Data Availability

No datasets were generated or analysed during the current study.
