# Supplementary material for: Bartonella in bat flies from the Egyptian fruit bat in the Middle East
Source: Parasitol Res. 2024 Feb 27;123(2):144. doi: 10.1007/s00436-024-08165-6 (PMC10899309; doi:10.1007/s00436-024-08165-6)
Supplement: Supplementary file 1 — Supplementary file1 (DOCX 21 KB) [file 436_2024_8165_MOESM1_ESM.docx]

**Table S1** Comparison of *Bartonella* sequences amplified from *Eucampsipoda aegyptia* with other sequences on GenBank via BLAST query

| ITS region | Country |  | OQ058984 | OQ058985 | OQ058986 | OQ058987 | OQ058988 | OQ058989 | OR523867 | OR523868 | OR523869 | OR523870 | OR523871 |
| --- | --- | --- | --- | --- | --- | --- | --- | --- | --- | --- | --- | --- | --- |
| B. rousetti KM382255 | Kenya | *Rousettus aegyptiacus* | 100% (311/311 bp) | 94.3% (246/261 bp) | 96.7% (289/299 bp) | 96.8% (214/221 bp) | 95.9% (235/245 bp) | 98.3% (228/232 bp) | 99.7% (355/356 bp) | 96.1% (323/336 bp) | 97.1% (338/348 bp) | 98.9% (354/358 bp) | 76.7% (240/313 bp) |
| B. sp. Lisso-Nig 922 MN504709 | Nigeria | *Myonycteris angolensis* | 100% (311/311 bp) | 94.3% (246/261 bp) | 96.7% (289/299 bp) | 96.8% (214/221 bp) | 95.9% (235/245 bp) | 98.3% (228/232 bp) | 99.7% (355/356 bp) | 96.1% (323/336 bp) | 97.1% (338/348 bp) | 98.9% (354/358 bp) | 76.7% (240/313 bp) |
| B. sp. NG13-090-1 MH142638 | Nigeria | *E. africana* | 97.1% (305/314 bp) | 93.7% (237/253 bp) | 93.7% (283/302 bp) | 96.9% (218/225 bp) | 94.7% (234/247 bp) | 97.4% (222/228 bp) | 97.2% (349/359 bp) | 97.6% (328/336 bp) | 99.7% (347/348 bp) | 96.4% (348/361 bp) | 77.0% (261/339 bp) |
| B. sp. NG13-078-1 MH142635 | Nigeria | *E. africana* | 96.8% (304/314 bp) | 93.3% (236/253 bp) | 94.4% (282/302 bp) | 96.4% (217/225 bp) | 93.9% (234/247 bp) | 96.2% (225/234 bp) | 96.9% (348/359 bp) | 98.2% (330/336 bp) | 99.1% (345/348 bp) | 96.1% (347/361 bp) | 76.4% (259/339 bp) |
| B. sp. NG13-013-1 MH142632 | Nigeria | *E. africana* | 99.7% (310/311 bp) | 93.9% (245/261 bp) | 96.3% (288/299 bp) | 96.4% (213/221 bp) | 95.5% (234/245 bp) | 97.8% (227/232 bp) | 99.4% (354/356 bp) | 95.8% (322/336 bp) | 96.8% (337/348 bp) | 99.2% (355/358 bp) | 76.4% (239/313 bp) |
| B. sp. strain R-255 MN258143 | Kenya | *Rousettus aegyptiacus* | 99.7% (310/311 bp) | 93.9% (245/261 bp) | 96.3% (288/299 bp) | 96.4% (213/221 bp) | 95.5% (234/245 bp) | 97.8% (227ú232 bp) | 99.4% (344/346 bp) | 95.8% (319/333 bp) | 96.8% (331/342 bp) | 98.6% (343/348 bp) | 77% (241/313 bp) |
| B. sp. MF288116 | Georgia | *Miniopterus schreibersii* | 87.3% (179/205 bp) | 92.2% (142/154 bp) | 92.2 % (142/154 bp) | 93.7% (133/142 bp) | 86.7% (170/196 bp) | 92.2% (142/154 bp) | 86.8% (178/205 bp) | 87.6% (169/193 bp) | 88.1% (170/193 bp) | 87.8% (180/205 bp) | 100% (24/24 bp) |
| B. tribocorum AF312505 |  |  | 93.8% (121/129 bp) | 93.8% (121/129 bp) | 93.8% (121/129 bp) | 93.8% (121/129 bp) | 93.0% (120/129bp) | 93.8% (121/129 bp) | 93.0% (120/129bp) | 93.0% (120/129bp) | 93.8% (121/129%) | 93.2% (123/132 bp) | 100% (23/23 bp) |
| B. grahamii AJ269789 | UK |  | 93.0% (120/129bp) | 93.0% (120/129bp) | 93.0% (120/129bp) | 93.0% (120/129bp) | 92.3% (119/129 bp) | 93.0% (120/129bp) | 92.3% (119/129 bp) | 92.3% (119/129 bp) | 93.0% (120/129bp) | 93.0% (120/129bp) | 100% (23/23 bp) |
| *gltA* gene |  |  | OR553951 | OR553952 |  |  |  |  |  |  |  |  |  |
| OR553951 |  |  |  | 76.2% (269/353 bp) |  |  |  |  |  |  |  |  |  |
| B. sp. YNBS/BF03 OP433671 | China | *E. africana* | 99.2 % (350/353 bp) | 76.6% (282/368 bp) |  |  |  |  |  |  |  |  |  |
| B. sp. YNBS/BF06 OP433673 | China | *E. africana* | 99.2% (350/353 bp) | 76.4% (281/368 bp) |  |  |  |  |  |  |  |  |  |
| B. sp. Batfly-3 LC461051 | Zambia | *E. africana* | 75.4% (245/325 bp) | 95.8% (319/333 bp) |  |  |  |  |  |  |  |  |  |
| B. sp. R-191 HM363764 | Kenya | *Rousettus aegyptiacus* | 85.0% (300/353 bp) | 73.0% (259/355 bp) |  |  |  |  |  |  |  |  |  |
| *B. bovis* LR15 MN615930 | Brazil | cattle | 90.9% (321/353 bp) | 75.2% (270/359 bp) |  |  |  |  |  |  |  |  |  |
| *B. melophagi* R-122-4 MT154631 | Peru | *Melophagus ovinus* | 90.4% (319/353 bp) | 74.6% (264/354 bp) |  |  |  |  |  |  |  |  |  |
| *B. clarridgeiae* BCF02 GU056189 |  |  | 90.4% (319/353 bp) | 75.0% (276/368 bp) |  |  |  |  |  |  |  |  |  |
| *B.* symbiont of *E. theodori* KT751156 | Comoros | *E. theodori* | 82.5% (273/331 bp) | 76.5% (254/332 bp) |  |  |  |  |  |  |  |  |  |
| *B. sp.* flyAA033  KR997986 | South Africa | *Eucampsipoda sp.* | 74.9% (179/239 bp) | 96.2% (229/238 bp) |  |  |  |  |  |  |  |  |  |
| *B. sp.* GLOSOR-HF16.1-94.1  MH234356 | Costa Rica | *Glossophaga soricina* | 88.6% (311/351 bp) | 77.4% (284/367 bp) |  |  |  |  |  |  |  |  |  |
